# Preliminary efficacy of a digital therapeutics smartphone application for methamphetamine use disorder: An experimental study

**DOI:** 10.3389/fpsyt.2022.1027695

**Published:** 2022-10-19

**Authors:** Liqun Zhang, Nan Li, Yuanhui Li, Tianjiao Zhang, Dai Li, Yanru Liu, Xiang Liu, Wei Hao

**Affiliations:** ^1^Adai Technology (Beijing) Co., Ltd., Beijing, China; ^2^Department of Industrial Engineering, Tsinghua University, Beijing, China; ^3^National Clinical Research Center on Mental Disorders, Mental Health Institute of the Second Xiangya Hospital, Central South University, Changsha, China

**Keywords:** digital therapeutics, methamphetamine, cognitive functions, approach bias modification training, cognitive behavioral therapy

## Abstract

Methamphetamine is the most widely used illicit drug in China. Treating methamphetamine use disorder (MUD) is challenging due to the lack of effective pharmacotherapies. This study is an experimental study to investigate the efficacy of smartphone-based digital therapeutics in treating MUD at the community level. One hundred participants were recruited and randomized into a digital therapeutics (DTx) group (*n* = 52) and a treatment as usual (TAU) group (*n* = 48). The DTx group used a smartphone application to deliver cognitive behavioral therapy, approach bias modification, cognitive training, and contingency management for 8 weeks. The TAU group received counseling from social workers and professional psychotherapists. Cue-induced craving, cognitive functions, PHQ9, and GAD7 were measured at baseline and post-intervention. Wilcoxon tests were performed with bootstrap and multiply imputation to estimate the treatment effect size. The DTx group showed a significant reduction in drug craving [Wilcoxon effect size = −0.267, 95% CI = (−0.435, −0.099), *p* = 0.002] and a significant improvement in cognitive function [Wilcoxon effect size = 0.220, 95% CI = (0.009, 0.432), *p* = 0.041]. The DTx group had overall 1, 8, and 24-week attritions of 8%, 11.5%, and 38.5%, respectively. The study shows that Digital therapeutics is feasible and potentially beneficial as a complement to community substance use treatment programs.

## Introduction

Methamphetamine is China's most widely used illicit drug, causing severe social, health, and economic problems and burdens. According to Chinese official statistics (1), an estimated 1.18 million methamphetamine users accounted for 55.2% of all drug users nationwide by the end of 2019. To battle illicit drug addiction, the anti-drug law of China was signed in 2007. The law mandates illicit drug users to undergo treatment programs (2).

Although effective treatments for methamphetamine use disorder (MUD) are urgently needed, pharmacotherapies have had modest effects so far (3–5). Due to the lack of effective pharmacotherapies, psychotherapies are considered a first-line treatment, with evidence showing that cognitive behavioral therapy (CBT) and contingency management (CM) reduce methamphetamine use (6–8). CBT is a form of “talk therapy” that can be used to teach, encourage, and support individuals to reduce/stop their harmful drug use (9). CBT is based on principles of conditioning and learning and provides valuable skills for gaining abstinence from drugs. CM is a behavioral technique based on the systematic application of principles of positive reinforcement (9). Approach bias modification (ApBM) is a cognitive training approach that aims to decrease automatically triggered impulses to approach drugs and drug-related stimuli (10, 11). ApBM is widely used in the treatment for alcohol use disorder. Research has shown that 4–12 ApBM sessions reduce alcohol relapse rates by 8–13% at 1-year follow-up (12–14).

However, most psychotherapies, including those mentioned above, require skilled social workers and therapists (9). Given the large MUD population in China, the lack of skilled psychotherapy workforce and inadequate financing make it challenging to treat MUD at the public health level, especially in underprivileged areas (15, 16). Moreover, the COVID-19 pandemic makes face-to-face psychological intervention more difficult (17). In addition, cognitive function, mainly working memory, is widely reported to be impaired in a MUD, which may contribute to the high relapse rate (18). Smartphone-based digital therapeutics (DTx) may address the above challenges by offering a ubiquitous and low-cost approach, which has been widely used in the intervention of substance addiction (19–21). These smartphone-based programs could enhance the reach of evidence-based interventions for populations with SUDs by delivering CBT, ApBM, cognitive training, and CM (10, 22).

In this study, we developed a smartphone application (WonderLab Harbor, Adai Technology (Beijing) Co. Ltd.) that combines CBT, ApBM, cognitive training, and CM to treat MUD at the community level. One hundred MUD participants were recruited from community-based rehabilitation programs in China. These programs are funded and operated by local municipalities and were the most widely adopted treatment schemes in China (23). One hundred MUD participants were recruited and randomized to a DTx group (*n* = 52) and a TAU group (*n* = 48) for 8-week DTx and TAU treatments. The baseline and post-intervention outcome measures included cue-induced craving, cognitive function scores, PHQ9, and GAD7. The DTx participants were allowed to use the DTx application after the program ended at week 8. We track and analyze the DTx users' activities within the DTx application for 40 weeks. This paper reports the findings and discusses the efficacy of smartphone-based digital therapeutics for treating MUD.

## Methods

### Study design

This study was a randomized experimental study to study the efficacy of digital therapeutics for treating MUD. Participants were randomly assigned to one of two 8-week treatment programs: 1. (DTX) digital therapeutics enabled rehabilitation and 2. (TAU) traditional community-based rehabilitation program (i.e., treatment as usual). The study was reviewed and approved by the participating community rehabilitation centers (funded and operated by the local municipalities) and was in accordance with the principles of the Declaration of Helsinki. The study is registered at clinicaltrials.gov (NCT05550493).

### Participants

Between January 2021 and March 2021, 100 participants diagnosed with MUD and about to undergo community-based rehabilitation were recruited voluntarily from four community-based rehabilitation centers in Chengdu, China. Inclusion criteria were: (1) age between 18 and 50 years, (2) meeting Diagnostic and Statistical Manual of Mental Disorders (DSM-IV) criteria for methamphetamine dependence. Participants who could not fluently operate an Android or an iOS smartphone were excluded. Participants who had mental health conditions other than MUD were excluded. Eligible participants were randomly assigned to the Digital Therapeutics (DTx) group (*n* = 52) or the Treatment As Usual (TAU) group (*n* = 48). The 8-week DTx and TAU programs started in March 2021 and ended 8 weeks later in June 2021.

### DTx intervention design

The DTx group was asked to download and install a smartphone application (WonderLab Harbor) that incorporated Internet-based Cognitive Behavioral Therapy (ICBT), Approach Bias Modification (ApBM), cognitive function training, and Contingency Management (CM). During the 8-week treatment program, the participants in the DTx group were instructed to complete ICBT, cognitive trainings, and ApBM trainings. Reward points (which can be redeemed for cellphone plan credit) were rewarded following completing each task as part of the positive reinforcement following CM principles.

#### Internet-based cognitive behavioral therapy

The DTx application delivers ICBT for treating MUD. The ICBT program consisted of eight interactive sessions, each requiring ~15 min to complete. Each ICBT session includes interactive multimedia modules (videos, pictures, and texts), the contents of which were based on the community reinforcement approach (24–26). The ICBT sessions covered the following topics: (1) introduction to digital therapeutics and CBT, (2) recognizing the triggers of craving, (3) coping with craving, (4) refusing skills/assertiveness, (5) problem-solving skills, (6) changing thoughts about drugs, (7) seemingly irrelevant decisions, and (8) HIV risks and prevention. Sessions were sequentially unlocked for participants upon completion. Participants could repeat any unlocked session as many times as they wished.

#### Approach bias modification

The DTx smartphone application also included an ApBM training module following the design in a previous ApBM design for reducing alcohol usage (27). In an ApBM session, users were instructed to swipe upward (downward) when they saw portrait (landscape) format images. A shrinking (growing) animation comes after swiping upward (downward) to simulate the visual effect of moving away (moving toward). The images were related to methamphetamine usage (methamphetamine crystals, powders, and paraphernalia) or healthy lifestyles (wealth, sports, gourmet, family activities, etc.). Each ApBM session was composed of presenting each one of the healthy lifestyle (methamphetamine) cues 12 times in landscape (portrait) and once in portrait (landscape). Hence, users were supposed to push away 92.3% of methamphetamine cues and pull 92.3% of healthy lifestyle cues toward themselves. Each ApBM session had 156 trials (6 healthy lifestyle cues repeated 13 times and 6 methamphetamine cues repeated 13 times) and took approximately 3 min to complete. The swipe direction and response time of each swipe were recorded. Extra reward points were given for correct and quick responses.

#### Cognitive function training

The DTx application incorporated a game-based cognitive function training module for improving working memory. In this game, a matrix of squares is displayed at the center of the screen. Between 3 and 5 target symbols are randomly placed in the matrix and displayed for 2 s. Next, the symbols disappear, and the matrix randomly rotates. The participants were asked to click the correct locations of the target symbols within the prescribed time limit. The game becomes more difficult as the size of the matrix, the number of target symbols, and the complexity of the matrix rotation change at each level. Each cognitive function training session lasted between 3 and 5 min.

#### Contingency management

Within the smartphone application, rewards were given as points to reinforce positive behaviors. When users logs-in to the DTx application, they can visit the daily check-in page to collect the daily login rewards. Users who login to the application consecutively can collect bonus points. In addition, points were given when the user unlocks an ICBT session (100 points) or completes an ApBM or a cognitive function training (50–100 points, depending on the user's performance). Within the DTx app, users can redeem points for cell phone plan credits. Every 1,000 points can be redeemed for a ¥ 10 CNY cell phone plan credit (approximately USD 1.6), which would cover 1/4 of a typical monthly cell phone plan. We did not set a limit for how many points could be earned and redeemed, and points did not expire (28).

### TAU intervention desgin

Upon enrollment, TAU participants were informed that they would receive weekly counseling sessions from social workers and professional psychotherapists. Due to COVID-19 pandemic control regulations, counselings were in the form of phone calls. The telephone counseling covered topics including work, family, stress management, and drug craving suppression. The social workers and psychotherapists will text or call the participants each week in the TAU treatment program to schedule a counseling session. The counseling telephone calls lasted 11 min on average.

### Outcome measures

The participants were required to complete a baseline assessment and a post-intervention assessment at week 8 to evaluate drug craving, depression and anxiety, and cognitive function.

#### Cue-induced craving

We used self-reported cue-induced craving scores as the primary outcome measures. We did not use toxicology test results since most toxicology screening results in the participating community-based rehabilitation programs were negative. All of our 100 participants had not had positive toxicology test results between January 2021 and June 2021, which made toxicology screening results unsuitable as an outcome measure.

The cue-induced craving was assessed by showing the participants images on a smartphone and asking them to rate their cravings on a 0–10 visual analog scale (0 being least craved and 10 being most craved). The images were related to methamphetamine (methamphetamine crystals, powders, and paraphernalia) or healthy lifestyles (wealth, sports, gourmet, family activities, etc.). These were the same images used in the ApBM module for DTx users. A total of 30 methamphetamine-related images and 30 health lifestyles images were assessed. The sum of the cravings for the 30 methamphetamine-related images was used as the drug craving score. Upon randomization, the DTx users were instructed to download and install the DTx application on their personal smartphones. Hence, the DTx users completed the craving assessments on their personal smartphones. The TAU group did not receive the download instruction. Hence, TAU users completed the craving assessment on-site using the social workers' smartphones within the DTx application. Between the baseline and post-intervention assessments, the TAU users did not have access to the DTx application.

#### Depression and anxiety

Participants completed the Patient Health Questionnaire-9 (PHQ9) and the Generalized Anxiety Disorder 7-item (GAD7) questionnaires on a smartphone to assess depression and anxiety.

#### Cognitive function

We use the Meaningless Figure Recognition Test (MFRT), a computerized visual memory test, to assess the cognitive function. This test has eight blocks, and each block has two phases. In the first phase, the participant is presented with a series of meaningless figures one by one for 3 s. The participants were asked to memorize these figures. In the second phase, the previously presented meaningless figures and the same number of the novel meaningless figures are presented on one screen in random order. The participants were asked to recall their memory and click all the previously presented figures within 15 s. The correct rate of clicks is used for measuring the cognitive function scores.

### Statistical analysis

All statistical analyses and visualizations were done in R 4.2.1.

#### Outcome measures response rate and imputation methods

The outcome measures did not have a 100% response rate in our study sample. Hence, some of the outcome measures were missing. To examine whether the missingness was missing at random, missing completely at random, or missing not at random, we examine the pattern of the missing measures and calculate the correlations between them.

We employed two sets of analyses to estimate the treatment effects on the outcome measures. The first used the complete cases (participants who responded at both baseline and post-intervention). The second used bootstrapped multiply imputed data by the Multivariate Imputation by Chained Equations (MICE) method (number of bootstrap samples = 2,000, and number of multiply imputed samples = 5) (29). The complete case analysis helps us estimate the treatment effects on participants who had a perfect response rate. The multiply imputed analysis helps us estimate the effects for the entire study cohort.

#### Treatment effects

We perform the Shapiro-Wilk test on the continuous variables to test for normality. The tests indicated that craving, PHQ9, GAD7, and age all exhibited non-normal distribution patterns (*p* < 0.001). The test on cognitive function scores indicated that it was likely to be normally distributed (*p* = 0.20). Due to the non-normality of most of our variables, we use the non-parametric paired Wilcoxon signed rank test to test the within-participant changes in each group. For the complete cases, the Wilcoxon effect sizes were calculated. For each estimate, bootstrap confidence intervals and bootstrap *p-*values were obtained using 2,000 bootstrap samples. For the multiply imputed analyses, we report the estimates of the Wilcoxon effect sizes, the 95% confidence intervals, and *p-*values.

### DTx application usage measures

DTx users' activities were logged in a database with timestamps. We analyze the user activities and attrition for 40 weeks after initial enrollment.

#### User activities

We calculate and plot the cumulative numbers of ICBT sessions, ApBM trainings, cognitive trainings, reward point redemptions, daily check-ins, and login days per user by week up to 40 weeks after initial enrollment.

#### User attritions

We identify the attrition point for each user for each DTx activity (ICBT sessions, ApBM trainings, cognitive trainings, reward point redemptions, and login). The attrition point is the time when the user stopped using the function and did not use the function after that. We calculate the cumulative number of attritions every week for 30 weeks after initial enrollment for each user. Hence, in week 30, if the user did not use the function in weeks 31 through 40, the attrition point is identified as week 30. Since the data is right censored, we did not identify attritions between weeks 31 and 40.

## Results

### Participants and response rate

Table 1 tabulates the participant characteristics by the group. Wilcoxon rank sum tests for the continuous variables found no significant difference between groups. Two-sample proportions tests (for sex) and Pearson's Chi-squared tests (for education and employment status) found no significant difference between groups.

**Table 1:** Participant characteristics and response rate.

**Variable^a^**	**DTx, *N* = 52^b^**	**TAU, *N* = 48^b^**	***p*-value^c^**
**Age**	38.28 (6.86)	38.07 (7.79)	0.804
**Sex**			0.482
Female	9/52 (17%)	5/48 (10%)	
Male	43/52 (83%)	43/48 (90%)	
**Employment status**			0.312
Employed	19/50 (38%)	13/46 (28%)	
Unemployed	31/50 (62%)	33/46 (72%)	
(Response rate)	50/52 (96%)	46/48 (96%)	
**Education**			0.531
Elementary school or below	6/33 (18%)	2/28 (7.1%)	
Middle school	21/33 (64%)	21/28 (75%)	
High school or above	6/33 (18%)	5/28 (18%)	
(Response rate)	19/52 (63%)	28/48 (58%)	>0.999
**Drug craving (baseline)**	5.86 (12.73)	3.86 (6.04)	0.995
(Response rate)^***^	46/52 (88%)	22/48 (46%)	<0.001
**Drug craving (post-intervention)**	2.89 (5.61)	6.31 (20.48)	0.858
(Response rate)^***^	31/52 (60%)	16/48 (33%)	<0.001
**PHQ9 (baseline)**	0.98 (2.07)	0.87 (1.62)	0.956
(Response rate)	50/52 (96%)	47/48 (98%)	>0.999
**PHQ9 (post-intervention)**	1.06 (2.01)	0.55 (1.06)	0.487
(Response rate)	33/52 (63%)	31/48 (65%)	>0.999
**GAD7 (baseline)**	0.66 (1.70)	0.43 (0.95)	0.735
(Response rate)	50/52 (96%)	47/48 (98%)	>0.999
**GAD7 (post-intervention)**	0.52 (1.00)	0.26 (0.51)	0.506
(Response rate)	33/52 (63%)	31/48 (65%)	>0.999
**Cognitive function score (baseline)**	71.51 (9.84)	73.38 (9.76)	0.427
(Response rate)	49/52 (94%)	48/48 (100%)	0.270
**Cognitive function score (post-intervention)**	75.97 (8.96)	73.62 (8.76)	0.367
(Response rate)	29/52 (56%)	29/48 (60%)	0.789

a,*** : p < 0.001.

b Mean (SD); n/N (%).

c Wilcoxon rank sum test; Pearson's Chi-squared test; Two sample proportions test.

As for response rates, two-proportion tests found that the DTx group had a significantly higher response rate for drug cravings measures both at baseline and post-intervention. Furthermore, the correlation analysis showed that the drug cravings at baseline and post-intervention were correlated (*r* = 0.84, *p* < 0.001); the drug cravings at baseline and the PHQ9 at baseline were correlated (*r* = 0.48, *p* < 0.001); the drug cravings at post-intervention and the PHQ9 at baseline were correlated (*r* = 0.59, *p* < 0.001); the drug cravings at baseline and the GAD7 at baseline were correlated (*r* = 0.56, *p* < 0.001); the PHQ9 at baseline and the GAD7 at baseline were correlated (*r* = 0.82, *p* < 0.001); and the PHQ9 at post-intervention and the GAD7 at post-intervention were correlated (*r* = 0.82, *p* < 0.001). Further, a one-way analysis of variance showed that the DTx group had a higher drug craving response rate at baseline (*F* = 25.81, *p* < 0.001); the participants who responded to the PHQ9 and GAD7 questionnaires at post-intervention had higher response rates in reporting drug craving at post-intervention (*F* = 40.19, *p* < 0.001); and the participants who responded the cognitive function tests at post-intervention had higher response rates in reporting PHQ9 and GAD7 at post-intervention (F = 51.22, *p* < 0.001). We had reasons to believe that the group difference primarily drove the missingness. Hence the missingness was likely missing at random. Leveraging the underlying correlations between the variables, the Multivariate Imputation by Chained Equations imputation method could be used to impute the missing values and obtain estimates for the entire cohort (29).

### Treatment effects estimates

Table 2 tabulates the estimates of the effect sizes across the four main outcome measures. The complete case analysis found that the DTx group had lower drug craving scores at post-intervention [Wilcoxon effect size = −0.402, *p* = 0.026, 95% bootstrap CI = (−0.638, −0.087)] and had higher cognitive function scores [Wilcoxon effect size = 0.312, *p* = 0.094, 95% bootstrap CI = (−0.047, 0.631)]. The PHQ9 and GAD7 did not show significant changes. The TAU group showed no significant changes across the four outcome measures.

**Table 2:** Treatment effect estimates.

**Group**	**Outcome Measure**	**Wilcoxon Effect Size^a^**	***p*-Value**	**Bootstrap estimate^a^**	**Bootstrap 95% CI**	**Bootstrap *p*-Value**	**Bootstrap MICE estimate**	**Bootstrap MICE 95% CI**	**Bootstrap MICE *p*-Value**
DTx	Drug craving	−0.402^**^	0.026	−0.396^**^	[−0.638, −0.087]	0.011	−0.267^***^	[−0.435, −0.099]	0.002
	Cognitive function Score	0.312^*^	0.094	0.312^*^	[−0.047, 0.631]	0.091	0.220^**^	[0.009, 0.432]	0.041
	PHQ9	0.009	0.979	0.005	[−0.334, 0.346]	0.971	−0.005	[−0.241, 0.231]	0.967
	GAD7	0.042	0.858	0.044	[−0.28, 0.39]	0.855	−0.011	[−0.244, 0.223]	0.930
TAU	Drug craving	−0.064	0.938	−0.029	[−0.718, 0.773]	0.966	−0.158	[−0.489, 0.172]	0.347
	Cognitive function Score	0.045	0.819	0.043	[−0.325, 0.395]	0.823	0.012	[−0.221, 0.245]	0.920
	PHQ9	−0.119	0.527	−0.116	[−0.447, 0.257]	0.511	−0.107	[−0.329, 0.114]	0.343
	GAD7	0.010	1.000	0.028	[−0.313, 0.415]	0.946	−0.101	[−0.311, 0.11]	0.349

a,* : p < 0.1;

** : p < 0.05;

*** : p < 0.01.

Using the bootstrapped multiply imputed data with Multivariate Imputation by Chained Equations (MICE), we estimate the effect size on the entire cohort. The DTx group had lower drug craving scores at post-intervention [Wilcoxon effect size = −0.205, *p* = 0.002, 95% bootstrap CI = (−0.435, −0.099)] and had higher cognitive function scores [Wilcoxon effect size = 0.220, *p* = 0.041, 95% bootstrap CI = (0.009, 0.432)].

### DTx usage

User activities within the DTx application were logged with timestamps in a database. We analyze the activities and attritions.

#### DTx user activities

**Figure 1:**
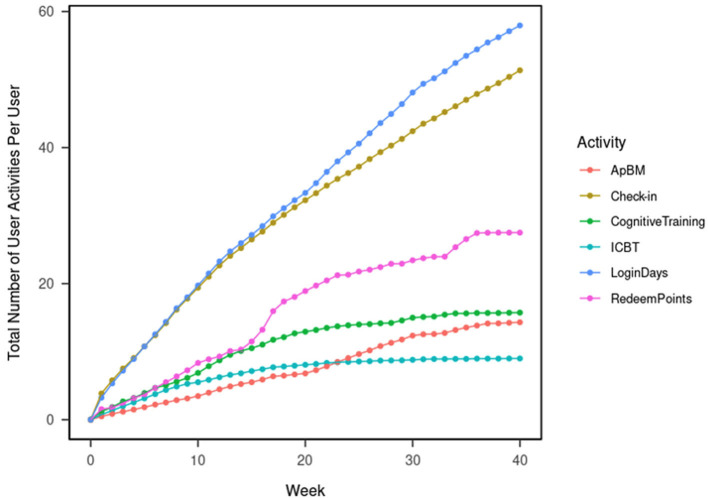
User activities in the DTx application.

Figure 1 shows the total number of DTx user activities logged in the database. This was calculated per user weekly over 40 weeks since the initial enrollment. In week 8 (which is the designed program duration and the time of post-intervention assessment), each one of the DTx users, on average, logged-in to the DTx for 16.37 days, completed 4.87 ICBT sessions, 5.58 cognitive trainings, 2.87 ApBM trainings, redeemed the reward points 6.35 times, and used the check-in function 16.17 times. Forty weeks after the initial enrollment, each one of the DTx users completed, on average, logged in to the DTx for 57.94 days, completed 8.98 ICBT sessions, 15.73 cognitive trainings, 14.31 ApBM trainings, redeemed the reward points 27.48 times, and used the check-in function 51.35 times.

The most-used functions were logins and daily check-ins. The least-used functions were ICBT, as the total number of ICBT activities plateaued in week 20. Overall, points redemption was more popular than cognitive training, followed by ApBM. After week 15, points redemption gained more popularity; after week 20, ApBM gained more popularity.

#### DTx user attrition

**Figure 2:**
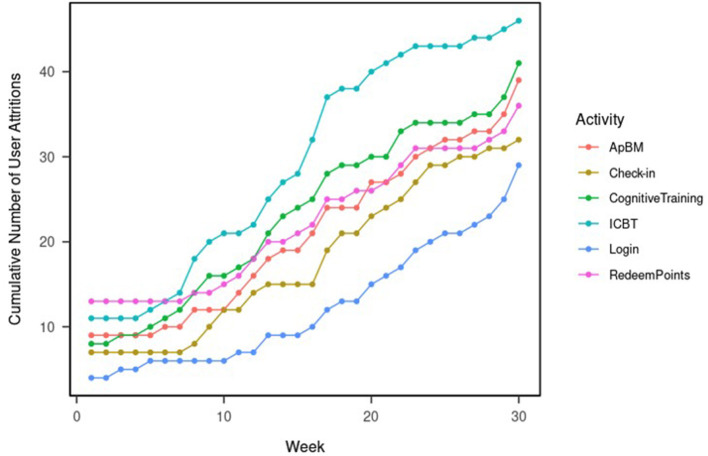
User attritions in the DTx application.

Figure 2 shows the cumulative number of user attritions. By the end of week 1, 11 users (21.2%) had stopped engaging in ICBT sessions, eight users (15.5%) had stopped doing cognitive trainings, 13 users (25%) had stopped redeeming points, nine users (17.3%) have stopped doing ApBM trainings, seven users (13.5%) have stopped using the daily check-in functions, and four users (8%) has completely stopped using the DTx application had never logged in again. The overall one-week attrition was 8%.

By the end of week 8 (post-intervention assessment time), 18 users (34.6) had stopped engaging in ICBT sessions, 14 users (26.9%) had stopped doing cognitive trainings, 14 users (26.9%) had stopped redeeming points, 12 users (23.1%) have stopped doing ApBM trainings, eight users (15.4%) have stopped using the daily check-in functions, and six users (11.5%) has completely stopped using the DTx application had never logged in again. The overall 8-week attrition was 11.5%.

By the end of week 24, 9 users (17.3%) were still viewing ICBT sessions, 18 users (34.6%) were still completing cognitive trainings, 21 users (40.4%) were redeeming points, 21 users (40.4%) were still doing ApBM trainings, 29 users (44.2%) were still using the daily check-in functions, and 32 users (61.5%) were still using the DTx application. The overall 24-week attrition was 38.5%.

## Discussion

To our knowledge, this is the first study to investigate the preliminary efficacy of a smartphone-based DTx intervention combining ICBT, CM, ApBM, and cognitive training principles in treating MUD at the community level in China. We discuss the design of the study, the efficacy, and the usage pattern of the DTx application.

### Study design and outcome measure response rate

The study randomized the 100 participants into the DTx and the TAU group. As Table 1 shows, the TAU group had a lower response rate in reporting cue-induced craving. We conjecture that this is because the TAU group were asked to complete the craving assessment on the social workers' smartphone. This meant that the randomization was not blinded. Moreover, the TAU users might have concerns with using the social workers' smartphones to report their cravings. This implied a limitation of our study. Future studies may consider designing and installing a “placebo” smartphone application for double-blinding.

### Treatment efficacy

This 52 vs. 48 experimental study showed preliminary evidence that the DTx application could benefit the treatment and rehabilitation of MUD at the community level with reduced cue-induced craving and improved cognitive functions. The findings support the growing evidence for the effectiveness of various digital psychosocial interventions used to treat substance use disorders (30, 31). Previous studies have demonstrated the clear efficacy of traditional psychosocial interventions, particularly CBT and CM, in treating MUD (6). However, these evidence-based treatments require well-trained and skilled therapists, who are sorely lacking in China, especially in underprivileged areas. Furthermore, drug users who receive regular community-based rehabilitation are reluctant to accept face-to-face interventions due to stigma. Digital therapeutics have the potential to address the above challenges by offering a ubiquitous, private and low-cost approach. In the last decade, various computer-assisted, internet-delivered, or smartphone-based digital interventions for substance abuse have been developed, most of which have demonstrated effectiveness (32–37).

### DTx usage and retention

Our results showed that the DTx group had overall 1-week attrition of 8%. In weeks 8 and 40, 88.5, and 44.2% of users were still using the DTx application, respectively. This showed that the DTx might have good adherence and retention. Our findings also showed that over the long run, the top used functions were check-in (for collecting reward points), points redemption, cognitive training, and ApBM. The least used function was the ICBT sessions. This showed that users were motivated by the rewards and willing to collect the “low-hanging fruits” (the daily check-ins in our DTx app).

Moreover, the cognitive and ApBM trainings gained more popularity over the long run because they are gamified and fun to play. ICBT sessions, however, consisted of only eight sessions. The low popularity was likely because rewatching the ICBT sessions provides diminishing utilities.

## Conclusions and limitations

This study was a randomized experiment to study the efficacy of using DTx to treat MUD at the community level. We found preliminary evidence that DTx could reduce cue-induced craving and improve cognitive functions. The limitations of the study are fourfold. First, the study is limited by its non-double-blinding and low response rates in the TAU group. Second, aside from cue-induced craving, we did not have the opportunity to use other outcome measures for drug craving and usage. Third, although the DTx participants' activities in the DTx application were monitored for 40 weeks and showed a high retention rate, we did not have the opportunity to follow up with the users at formal encounters to measure long-term outcomes. Fourth, the sample size of 100 is relatively small. Fifth, the design of the TAU treatment schemes could be further enhanced.

## Data availability statement

The original contributions presented in the study are included in the article/supplementary materials. Due to the nature of this research, participants of this study did not agree for their data to be shared publicly, so supporting data is not available. Further inquiries can be directed to the corresponding author.

## Ethics statement

The studies involving human participants were reviewed and approved by National Clinical Research Center on Mental Disorders and Mental Health Institute of the Second Xiangya Hospital, Central South University, Changsha, China. The patients/participants provided their written informed consent to participate in this study.

## Author contributions

LZ: conceptualization, writing—original draft preparation, investigation, data curation, and software. NL: data curation, formal analysis, and writing—review and editing. YLi: conceptualization, validation, and software. TZ: data curation and software. DL: conceptualization and project administration. YLiu: writing—original draft preparation and writing—review and editing. XL: conceptualization, formal analysis, validation, and supervision. WH: supervision and writing—review and editing. All authors contributed to the article and approved the submitted version.

## Conflict of interest

Authors LZ, YLi, TZ, DL, and YLiu were employed by Adai Technology (Beijing) Co., Ltd., Beijing, China. Authors NL and XL are supported by the Natural Science Foundation of China Grant 72001122.

The remaining authors declare that the research was conducted in the absence of any commercial or financial relationships that could be construed as a potential conflict of interest.

The authors declare that this study received funding from the Natural Science Foundation of China (Grant No. 72001122) and Adai Technology (Beijing) Co., Ltd., Beijing, China. The funders had the following involvement with the study: study design, data collection, and analysis.

## Publisher's note

All claims expressed in this article are solely those of the authors and do not necessarily represent those of their affiliated organizations, or those of the publisher, the editors and the reviewers. Any product that may be evaluated in this article, or claim that may be made by its manufacturer, is not guaranteed or endorsed by the publisher.
